# Diagnosis of Hypertrophy of the Brain from Chronic Hydrocephalus

**Published:** 1849-02

**Authors:** 


*Diagnosis of Hypertrophy of the Brain from Chronic Hydrocephalus.*—In a review of M. Mauthner's work on Diseases of the Brain and Spinal Marrow in Children, in the British and Foreign Review, the following propositions, tending to illustrate this doubtful point, are collected:

*Hypertrophy of the Brain.*—1. The posterior part of the skull first presents an unnatural prominence.

2. Children lie horizontally, or throw the head back.

3. Face puffy, eyes inexpressive and staring, mouth half open.

4. Functional disturbance comes on very gradually—not before the period of dentition or weaning—and consists at first in affection of the respiratory apparatus, difficulty of breathing, and attacks of apnœa.

5. Patient fat and leucophlegmatic.

*Chronic Hydrocephalus.*—1. The forehead is the first part to present unnatural prominence; the altered direction of the eyes, and the very great width of sutures and fontanelles are likewise characteristic.

2. Children lie on the belly, with the head lower than the rest of the body, burying the face in the pillow.

3. Countenance withered, having expression of premature old age.

4. Functional disturbance occurs early, and involves the cerebrum from the very beginning.

5. Patient ill-nourished, subjects to rickets and tabes mesenterica.—*American Journal and Library of Dental Science.*

